# Perceptions of rural primary healthcare personnel about expansion of early communication intervention

**DOI:** 10.4102/phcfm.v5i1.553

**Published:** 2013-10-25

**Authors:** Jeannie van der Linde, Alta Kritzinger

**Affiliations:** 1 Department of Communication Pathology, University of Pretoria, South Africa

## Abstract

**Background:**

Early communication intervention services rendered by speech-language therapists and audiologists to families of infants and young children with feeding difficulties, hearing loss or emerging communication disorders should be implemented throughout South Africa. Early intervention can ameliorate risks, enhance development and may prevent further delays. Based on research initiated during a community-service year experience in a rural subdistrict, an incremental process of establishing accessible early communication intervention services was deemed feasible. Such a process cannot be successful if the collaboration of primary healthcare personnel and managers is not ensured.

**Objectives:**

The aim of the article was to describe the perceptions of primary healthcare personnel with regard to expansion of early communication intervention services to infants at risk of developmental delay.

**Method:**

A qualitative descriptive survey design was followed. Semi-structured interviews were conducted with 20 primary healthcare nurses and sisters and eight primary healthcare programme managers in Ditsobotla subdistrict in the North West province of South Africa.

**Results:**

The participants indicated that by improving team work, developing training programmes and evaluating identification methods and resources, the step-by-step rollout of early communication intervention functions on four organisational levels may be a realistic goal for sustainable services in the resource-limited district.

**Conclusion:**

The positive perceptions and contributions by participants promise a rich human-resource basis for transdisciplinary collaboration between speech-language therapists, audiologists and primary healthcare personnel in order to reduce the burden of early communication disorders in a rural district.

## Abstrait

**Les opinions du personnel de soins de santé primaires sur le développement des interventions en troubles précoces de la communication**

**Contexte:**

Les services d'intervention en troubles précoces de la communication fournis par les orthophonistes et audiologistes aux familles de nourrissons et jeunes enfants présentant des difficultés d'alimentation, une perte auditive ou des débuts de troubles de la communication devraient être mis en place dans toute l'Afrique du Sud. L'intervention précoce peut limiter les risques, améliorer le développement et contrer des retards supplémentaires. Sur la base d'un travail de recherche entrepris pendant une expérience d'un an de service communautaire dans un sous-district rural, un processus progressif d’établissement de services accessibles d'intervention en troubles précoces de la communication a été jugé faisable. Un tel processus ne peut réussir si la collaboration du personnel et des cadres de soins de santé primaires n'est pas assurée.

**Objectifs:**

Le but de l'article était de décrire les opinions du personnel de soins de santé primaires quant au développement des services d'intervention en troubles précoces de la communication chez les nourrissons à risque de retard du développement.

**Méthode:**

Un modèle d’étude descriptive quantitative a été suivi. Des entretiens semi-structurés ont été conduits avec vingt infirmiers/-ières et soeurs et huit responsables de programme de soins de santé primaires dans le sous-district de Ditsobotla, dans la province sud-africaine du Nord-Ouest.

**Résultats:**

Les participants ont indiqué qu'en améliorant le travail d’équipe, en développant des programmes de formation et en évaluant des méthodes et ressources d'identification, le déploiement étape par étape de fonctions d'intervention en troubles précoces de la communication à quatre niveaux organisationnels pouvait être un objectif réaliste pour des services durables dans ce district aux ressources limitées.

**Conclusion:**

Les opinions et contributions positives des participants laissent promettre une base fournie de ressources humaines pour une collaboration transdisciplinaire entre les orthophonistes, les audiologistes et le personnel de soins de santé primaires afin de réduire le poids des troubles précoces de communication dans un district rural.

## Introduction

Early communication intervention, an evidence-based approach to the comprehensive management of feeding difficulties, hearing impairment and emerging communication disorders in infants and young children, is now established at most tertiary-level public hospitals and private practices in South Africa. Training in early communication intervention is included in all undergraduate Speech-Language Pathology and Audiology programmes.^1^ Despite the lack of public policy, early communication intervention as a subject field and a service is expanding in South Africa. Since the turn of the millennium local publications have suggested strategies for community-based implementation of early communication intervention in Primary Health Care (PHC).^2, 3^ A renewed call for the establishment of early communication interventions in rural communities was made by the South African Speech–Language–Hearing Association (SASLHA).^4^ The implementation of early communication intervention services in PHC and community-based contexts is limited.^5^ The lack of clear procedures, evidence-based practice guidelines and health policy on how to implement early communication intervention in PHC, as well as the fact that early communication intervention is an unknown service amongst most healthcare professionals and the public in general, may be some of the reasons why few speech-language therapists and audiologists respond to the challenge to establish formal community-based early communication intervention programmes.

Establishing a comprehensive early communication intervention programme in a rural area is a large undertaking, but a step-by-step approach to implementation may be a feasible option.^6^ The rationale for an incremental implementation of early communication intervention functions is found in resource limitations and the needs of infants in rural communities in South Africa.

As the main concentration of families living in poverty is found in rural areas, poverty should be regarded as a key risk factor influencing infant development in rural communities.^7^ Of the 18.7 million children in South Africa, the majority are living in provinces with large rural populations, such as Limpopo, the Eastern Cape and KwaZulu-Natal.^8^ It is generally accepted that children living in poverty are at increased risk of developing disability.^9^ Due to the prevalence of risk conditions such as HIV, foetal alcohol spectrum disorder, prematurity, low birth weight and cerebral palsy in South Africa,^2^ infants and young children are at an even higher risk of hearing loss, feeding and communication disorders. A great need for early interventionists to focus on prevention of communication disorders is therefore indicated.^2, 10^ A careful look at the evolution of PHC as implemented by trained personnel and managers in South Africa may provide strategies for the much-needed implementation of all early communication intervention services in rural communities.

PHC is one of the five health priorities for South Africa and the Minister of Health recently called for a renewed emphasis on prevention of disease instead of a curative approach to healthcare.^11^ Since the implementation of PHC in the South African health system, communities and individuals who previously did not have access to health services have reaped numerous benefits from the system. According to the PHC Facilities Surveys of 1998 and 2000, significant improvements, such as better emergency-vehicle response times, daily immunisation opportunities for infants and children and antenatal services, are now evident in both rural and urban areas.^12^ Currently, however, due to a largely hospital-centric healthcare system, epidemics, poor partnering with communities and poor multisectoral collaboration, comprehensive and integrative primary care were not realised to the majority of South Africans.^13^ These challenges resulted in passive, poor-quality and sporadic PHC services, whereas the hospitals were overburdened with referrals and patients in need of services.^13, 14^ The National Department of Health^15^ responded to these challenges with the ‘Revitalisation of Primary Health Care’ initiative as a means of re-engineering PHC toward proactive home- and community-focused interventions. As a result, municipal ward outreach teams, district specialists’ teams and school health services^13, 15^ should be integrated into the PHC Package.

Originally, the PHC Package^16, 17^ was designed to adapt the previous health system through comprehensive and integrated services and can therefore not be implemented through separate, vertical programmes by personnel not collaborating with one another.^12^ Vertical programmes with a narrow focus often split services according to disciplines, thereby hindering teamwork across different professions.

As indicated in Table 1, the PHC Package^16, 17^ is a standardised, comprehensive ‘basket’ of services, including preventive-, promotive-, basic curative- and rehabilitative services delivered at community level. The package stipulates the common quality norms and standards for each PHC service and should receive mutual support from healthcare professionals delivering the services.^16^ A ‘one-stop’ approach is facilitated where interventions are delivered in clusters, congruent with the infrastructure and the model of care at district level.^12^ PHC facilities in South Africa had already been identified by audiologists as viable platforms for the early identification of hearing loss.^18^

**TABLE 1:** Components of the Primary Health Care Package.

PHC Programmes	Description
1. Non-personal health services	Promotion of occupational health Promotion of health and disease prevention Dissemination of information on environmental health
2. Disease prevention and control	Prevention of chronic diseases Promotion of well-being in geriatric patients Rehabilitation of disabilities Promotion of oral health and prevention of oral diseases Prevention of communicable diseases (including notifiable medical conditions)
3. Maternal, child and women's health	Provision of ante-natal and post-natal care Provision of contraceptive methods Screening for cervical cancer Termination of pregnancy Provision of genetic services Management of childhood illnesses and immunisation Intervention in protein-energy malnutrition Sustaining the primary-school nutritional programme
4. HIV, sexually-transmitted infections and tuberculosis	Support and home based care for HIV patients Voluntary confidential counselling and testing for HIV Prevention of mother-to-child HIV transmission Prevention and management of sexually-transmitted diseases Diagnosis and treatment of tuberculosis
5. Health monitoring and evaluation	Obtaining health information from healthcare facilities Surveillance of the public-health system Coordination of research on health-related topics and current issues influencing healthcare in South Africa
6. Mental health and substance abuse	Prevention and treatment of mental disability Prevention of substance abuse
7. Gender issues	Referral and counselling of victims of violence and sexual abuse
8. Municipal ward outreach teams	Collective facilitation of community involvement and participation in identifying health problems and behaviours that place individuals at risk of disease or injury Identify vulnerable individuals and groups Implementation of appropriate interventions from the service package to address the behaviours or health problems
9. District-specialist teams	To promote innovative models of providing specialist healthcare closer to the patients’ homes To promote integrated working practices between general practitioners and hospital-based specialists. To improve the quality of services rendered at the first level of care by ensuring adherence to treatment guidelines and protocols To provide peer support for specialists working in primary healthcare
10. School health teams	Health promotion, prevention and curative health services that address the health needs of school-going children, including those children who have missed the opportunity to access services such as child immunisation services during their pre-school years

*Source*: National Department of Health 2011 15; Department of Health 200117

When viewing the PHC Package with its 10 different programmes in Table 1, it is clear that early communication intervention services should be offered when mothers or caregivers of infants and young children visit a health facility. An early communication intervention programme should therefore be integrated into several PHC programmes and not offered as a vertical programme dependent on one discipline only, such as Speech-Language Pathology or Audiology.

The onset and progression of communication disorders may be reduced or eliminated by changing the susceptibility or minimising exposure to the risk factors that influence prenatal- and postnatal development in infants and young children.^19, 20^ It is therefore evident that early communication intervention operates mainly on the primary- and secondary levels of prevention. Both early communication intervention^21^ and the PHC approach stipulate that programmes should address the specific needs of communities, with full participation of families and community members. Services should be accessible to all, affordable, culturally acceptable and implement appropriately-selected evidence-based procedures. Both PHC and early communication intervention are comprehensive, should be well coordinated with partners and should be based on teamwork.^4, 12, 22, 23^

Based on research initiated during a community-service year in Ditsobotla subdistrict in the North West province of South Africa^6^ and as reported in van der Linde et al.,^5^ the full-scale implementation of all early communication intervention functions at the PHC facilities in the subdistrict was neither possible nor sustainable. The study found that the identification methods for infants at risk of communication delay were limited and unreliable and that the referral system in the subdistrict was ineffective.^5^ An incremental rollout of the different early communication intervention functions such as promotion of normal development, developmental surveillance of infants at risk, assessment, providing intervention and parent training was suggested. As the implementation of the different functions depends on the collaboration between a speech-language therapist or audiologist, as well as the PHC personnel at each health facility and their managers, it is important to investigate perceptions and attitudes toward the proposed plans. The article presents the perceptions of rural healthcare workers to implement sustainable early communication intervention programmes in remote PHC facilities so that accessible and best-practice services can be provided to infants at risk and their families.

### Objectives

The purpose of this article is to describe the perceptions of PHC personnel with regard to the expansion of early communication intervention services to infants at risk in the rural subdistrict of Ditsobotla in South Africa. The following objectives were pursued:

- To describe the participants’ perceptions regarding case finding, identification methods, resources and limitations in Ditsobotla subdistrict.
- To describe the self-identified training needs amongst PHC personnel and managers for early communication intervention in the subdistrict.
- To describe the team approach suggested by the participants.

## Research method and design

A qualitative descriptive study was conducted as the researchers attempted to gain a first-hand and holistic understanding of the perceptions of participants.^24^ Semi-structured interviews with the PHC personnel and programme managers of Ditsobotla subdistrict were conducted. The two data sources, namely those of the PHC personnel and the programme managers, served as data triangulation which increased the reliability of the study. The purpose was therefore to shift the research from an investigator perspective to that of the participants and to report on the qualitative data which were collected.^6^

### Research context

**FIGURE 1:**
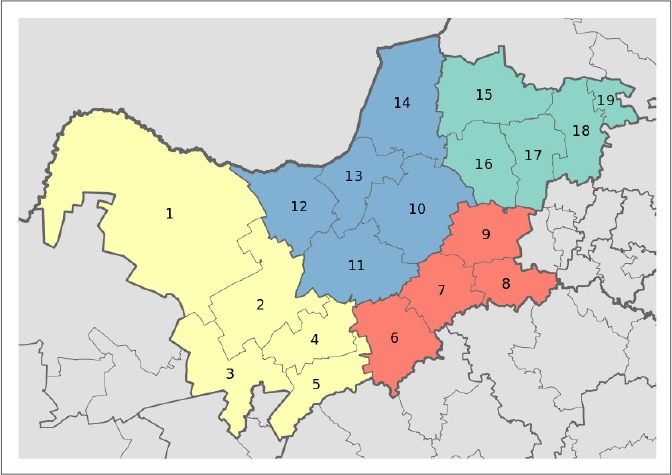
Area map of the North West province. Ditsobotla subdistrict is situated at the centre of the province and is represented by the numeral 10. *Source:* http://en.wikipedia.org/wiki/File:Map_of_the_North_West_with_districts_shaded_and_ municipalities_numbered_(2011).svg

The subdistrict forms part of the central district in the North West province of South Africa and is characterised by small communities, mostly Setswana speaking. The subdistrict covers an area of 6465 square kilometres (2496.2 square miles), with a population density of 59.1 people per square mile. The largest town in the area is Lichtenburg. Income is generated by the agricultural activities of private landowners, mining and tourism, but many families rely on subsistence farming on ancestral land allocated by the tribal chief.^25^ The first researcher was stationed at the Lichtenberg hospital as a community-service speech-language therapist and audiologist. Public-health services in the subdistrict were organised according to the PHC model, with 17 clinics and three small hospitals. The travelling distances between Lichtenburg and the different clinics range from 3 km to 35 km, allowing a clinician to visit two clinics per day. A map highlighting the research setting can be seen in Figure 1.

The PHC clinics and hospitals in Ditsobotla subdistrict were selected randomly from two strata. The clinics were subdivided in the strata according to the size of the clinic: small clinics such as eight-hour clinics and mobile clinics were placed in stratum 1 and large clinics such as 24-hour PHC centres and district hospitals were placed in stratum 2. Twenty participants were recruited from the randomly-selected facilities, simply by asking the PHC personnel (with support from the PHC facility manager) who would like to participate as the participants in Group 1. The only exclusion criterion was little or no verbal competence in English or Afrikaans, which were the researchers’ primary languages, meaning that all prospective participants were competent in either Afrikaans or English. In Group 2, all 12 PHC programme managers of Ditsobotla subdistrict were invited to participate in the research.

### Participants

All the participants (PHC personnel and programme managers) had to be employed in Ditsobotla subdistrict and proficient in English in order to participate in the study. Approximately one-third (20) of the PHC personnel (Group 1) in the entire subdistrict were included in the study, with each stratum represented equally. The characteristics of the participants in Group 1 are summarised in Table 2. The majority of the participants had nursing diplomas and all were working on a full-time basis, with an average of eight to 12 years’ work experience. Although three participants had no formal training, their work experience rendered them as informed research participants.

**TABLE 2:** Characteristics of the primary healthcare personnel (*n* = 20).

Characteristic	Stratum 1 (*n* = 10)	Stratum 2 (*n* = 10)
Qualification	B Cur Degree: 2 Nursing Diploma: 6 No formal training: 2	B Cur Degree: 3 Nursing Diploma: 6 No formal training: 1
Years of experience	4 years – 25 years Average: 12,5 years	1 year –26 years Average: 8,4 years
First language	Setswana: 10	Setswana: 9 Afrikaans: 1
Employment status	Full-time: 10	Full-time: 10
Type of facility where employed	8-hour clinic: 7 12-hour clinic: 3	PHC hospital: 2 24-hour clinic: 8

Participants in Group 2 represented 67% of the PHC programme managers in the subdistrict (Table 3). According to Table 3, most of the programme managers had qualifications and work experience. A variety of programmes in the PHC package were represented by the managers, but those who managed the Maternal, Child and Women's Health, Mental Health and HIV programmes could not be included as participants as they were attending a course during the time allocated for data collection.

**TABLE 3:** The characteristics of the primary healthcare programme managers (*n* = 8).

Characteristics	Values
Qualifications	Masters degree: 1 B Cur degree: 2 Nursing diploma: 4 No formal training: 1
Experience in programme managing	Range: 1–10 years Average: 4 years
Facilities visited by primary healthcare programme managers	Hospitals, clinics, frail-care centres, prisons, hospices and schools, crèches and business premises
First language	2: Afrikaans 6: Setswana
Employment status	All full-time
Primary healthcare programme managed	Geriatrics, chronic diseases and rehabilitation Nutrition Communicable diseases Occupational therapy Health promotion School health Environmental health

### Material

Since face to face interviews have the highest response rate in survey research,^26^ semi-structured interviews were used to collect the data from both groups of participants. The different open-ended questions explored the following needs and perceptions of the participants: their information needs about early communication intervention and how case finding, the referral system and collaboration can be improved. The interviews began with an explanation of early communication intervention, followed by the following question: ‘Speech-language therapists and audiologists are experiencing difficulty in finding babies who need speech and hearing services in the rural areas. How can we work together to address the problem?’

### Procedures

In order to increase the credibility of the data and to test the two interview schedules, two pilot studies were conducted. Two PHC nurses and one PHC programme manager were selected to be part of the pilot studies. The interview schedules were adapted according to the results obtained in the pilot studies.

During the visit to each of the randomly-selected facilities the facility manager explained the reason for the visit and introduced the researcher to potential participants in Group 1. Informed consent was obtained from the participants and the interviews were conducted in consultation rooms at the different PHC facilities. The researcher visited the participants in Group 2, the programme managers at the district office of Ditsobotla. Informed consent was obtained and the semi-structured interviews were conducted in each participant's office. The arrangement made the participants feel comfortable and communication barriers were limited as a result of the professional environment in which the interviews were conducted.

### Data analysis

The interviews were recorded and the digital voice files were transcribed. A second reviewer was utilised in order to verify the interview transcripts of both groups of participants. The reviewer listened to the recordings whilst reading the transcriptions. Discrepancies between the text- and voice recordings were discussed until agreement was found between the researcher and second reviewer. The transcripts were read holistically and then re-read before the corpus was categorised according to themes. Regularities, or themes, were noted during the data analysis and categories of meaning emerged that could be presented as descriptive results. As two data sources were included in the data collection (data from the PHC personnel and the programme managers) common themes across the two data sets confirmed the reliability of the data and highlighted the categories of meaning that had been identified.

## Results

### Objective 1: Identification methods and resources in Ditsobotla subdistrict

Useful findings regarding specific resource needs to identify infants at risk were obtained and are paraphrased below:

- PHC personnel reported that they were not successful in identifying infants and young children at risk of developmental delay in the subdistrict.
- No reliable and valid identification methods and instruments for hearing loss, feeding difficulties and communication delay in infants and children were being used.
- PHC personnel indicated that parents, grandparents and other caregivers are either barriers or helpful resources when the developmental history of infants and young children has to be collected at clinics.

It is therefore imperative that a standardised screening instrument for risks of communication delay should be developed. Apart from the lack of screening instruments, another limitation is the lack of information on developmental history of the infant or young child obtained from parents, grandparents and caregivers during immunisation. As information on children's disabilities may not be shared by parents due to shame or other reasons, grandparents and caregivers may not know the case histories when bringing infants for immunisation. Despite these challenges, caregivers remain a valuable source of information about the children in their care and should be utilised. Research at the Clinic for High Risk Babies (CHRIB), University of Pretoria, found that parents consistently identified their children's communication difficulties earlier than professionals even though they might not act upon their concerns.^2^ Parents from all cultures strive to help their children to develop maximally, but they require knowledge to support their children's development.^10^ Consequently, parents, caregivers and grandparents need training,^27^ as they are irreplaceable members within the early communication intervention team. It is therefore essential to develop a training package for families of infants at risk.

### Objective 2: Perceptions on training

#### Subtheme 1: Professional training programmes

The responses of two PHC programme managers, when asked what information they need regarding early communication intervention, early identification of delays or disorders and the referral process, were:

‘[*We need*] brochures, information booklets on general services, signs and symptoms and how to go about caring for that person with the problem’. (Manager, female, 40 – 45 years old)

‘[*Training*] to indicate problems or signs; what is it that shows communication problems in children and what steps to take thereafter’. (Manager, female, 50 – 55 years old)

The information needs expressed by the majority of the healthcare participants, along with their willingness to receive information, imply that an early communication intervention information package must not only be developed for families but for professional collaborators as well. Training is required so that healthcare partners can assist in the identification and referral of infants and young children at risk for communication disorders, hearing loss and feeding difficulties. Training has to be provided to all PHC personnel, programme managers and managers of the facilities.

#### Subtheme 2: Promotional activities

Two of the PHC programme managers responded with the following statements, when asked why parent and caregiver training on communication and literacy development in young children should be integrated in PHC:

‘To make mothers aware. They are twenty-four hours with the child and they can detect and refer early, then there is less damage’. (Manager, female, 40 – 45 years old)

‘Mothers spend most of their time with the child, they know and can really identify. We only see the child for twenty minutes’. (Manager, female, 45 – 50 years old)

It is therefore evident that appropriate information should be provided to the community by means of talks and workshops to caregivers who attend immunisation clinics, as well as mass-communication media. Since the aim of mass media strategies is to educate the public, shape public behaviour and advocate services,^28^ posters and pamphlets on child development and other topics should be developed in the local languages. Popich et al.^10^ describe the development of an educational DVD for caregivers as a strategy for primary prevention of communication disorders in a specific community in South Africa. It is suggested that parents, grandparents and other stakeholders from the community should be involved in the planning of such an informational tool in order to ensure that their needs and values are reflected in the final product.^10, 27^

### Objective 3: Team approach for early communication intervention in Ditsobotla subdistrict

The team approach suggested by the participants of both groups is presented according to six subthemes that became evident during data analysis.

#### Subtheme 1: Collaborative partnerships amongst communities and professionals in primary healthcare

In response to a question regarding the advantages of collaboration for PHC programmes in Ditsobotla subdistrict, two of the PHC programme managers replied:

‘We will work collaboratively and other programme managers will know about early communication intervention services. You will get patients from all angles since all the PHC managers are aware’. (Manager, female, 40 – 45 years old)

‘We get a well-informed community, personnel and volunteers. We get a healthy community, because they will be informed’. (Manager, female, 45 – 50 years old)

The quotes reflect the perception that there is a close relationship between case finding, effective service delivery and collaboration. According to the participants, a clear advantage of collaboration would be a greater awareness of early communication intervention amongst health workers and the community. The participants also indicated that collaboration is limited in the subdistrict and the following suggestions were made to improve team work:

- An information sheet should be made available about the specialised services offered by early communication intervention.
- Trusting relationships between the speech-language therapist or audiologist and PHC personnel should be developed.
- Positive attitudes toward the newly-established early communication intervention services should be created.
- The permanent employment of a speech-language therapist or audiologist within the subdistrict is critical to the process.

The professions and the concept of early communication intervention must therefore be introduced to the PHC facilities and communities. The speech-language therapist or audiologist should be the leading professional in the collaborative process of establishing early communication intervention.

#### Subtheme 2: Building a comprehensive team

One of the PHC personnel responded with the following statement, when asked why team work will influence early identification and referral of infants positively:

‘As a team it makes work easier – quality nursing care. I can be taught some things, but the specialist [*speech-language therapist*] can do some work [*i.e. rendering of early communication intervention services*]. The team will provide better quality care’. (Manager, female, 45 – 50 years old)

The participants indicated that other healthcare professionals, such as occupational therapists, physiotherapists and dieticians should also be involved. Similar to the speech-language therapist or audiologist, many of these professionals are also annual community-service professionals and their services may be disrupted when clinicians are replaced or their posts are discontinued after a year.

Further results indicated that the teamwork in Ditsobotla subdistrict was uncoordinated, although different professionals are available. The PHC personnel only consulted with doctors during their visits to the facilities.

#### Subtheme 3: The role of primary healthcare programme managers

The PHC programme managers are a resourceful group of professionals who can assist the speech-language therapist or audiologist in the implementation of early communication intervention services in a rural community. When two of the PHC programme managers were asked to comment on how they see the future of working in collaboration speech-language therapists or audiologists, they responded:

‘Services will be marketed; it will be cost effective because we are working together, for example with transport. The PHC programme managers are from the same culture [*as the community members]* and they know the language and therefore they may help the clinician. Services will be well known to the community’ (Manager, female, 50 – 55 years old)

‘It is easier for the PHC programme manager to reach more people; fieldworkers get to more people, know the professions better, managers know what to do in case finding’. (Manager, female, 50 – 55 years old)

It is therefore evident that the PHC programme managers can assist speech-language therapists to reach the public, as they have already established a relationship of trust with the communities. Language- and cultural barriers can be overcome with the support of the PHC programme managers as they often share the same cultural backgrounds and speak the same languages as the local communities.^6^ The PHC programme managers’ knowledge and status in the community should be regarded as an asset and partnerships should be established in order to formalise early communication intervention in PHC. Programme managers are invaluable partners who can support the clinician in planning and implementing collaborative early communication intervention activities and integrating the services into the different PHC programmes. As managers, they have more authority than PHC personnel and can therefore exert more influence to assist with case finding.

#### Subtheme 4: Recruiting volunteers from communities

As speech-language therapists and audiologists are burdened with large caseloads in SA, it may not be possible to successfully fulfil all the functions included in the scope of early communication intervention practice. A PHC programme manager in Ditsobotla subdistrict recommended the following:

‘Speech-language therapists should recruit volunteers. They [*volunteers*] can play a supportive role and can help [*the clinician*] in overcoming language and cultural barriers’ (Manager, female, 45 – 50 years old)

If trained, volunteers can play a supportive role during the implementation and management of early communication intervention services in communities.

#### Subtheme 5: The suggested formalised process

The participants recommended that various aspects be taken into account in the implementation of early communication intervention services. The results were summarised as follows:

- The process should commence with meetings and negotiations with the health managers of the subdistrict. Starting at entry level, collaboration should therefore be the vehicle throughout the process of implementation.
- Establish a good relationship with management, namely, the Assistant Director of Community Health services in Ditsobotla subdistrict.
- Other community stakeholders, such as churches and religious communities, crèches and youth centres, could also be part of the planning and implementation of collaborative early communication-intervention functions.
- Continued and once-off collaborative activities should be planned in partnership with the PHC programme managers as these will improve the accessibility and affordability of services. The speech-language therapist and audiologist should manage early communication intervention as a PHC programme and should therefore play a promotional as well as an educational role in the developmental phase of early communication intervention services and thereafter.

**FIGURE 2:**
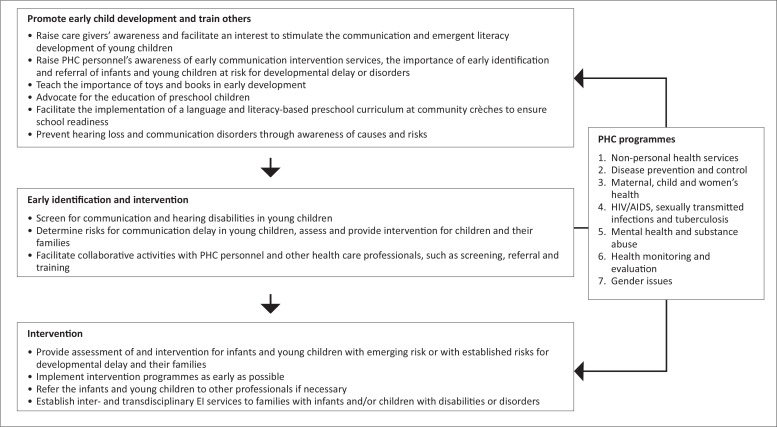
Early communication-intervention functions according to the levels of prevention in primary healthcare. *Source:* Department of Health 2001 ^17^; Kritzinger & Louw 2003 ^21^; Feldman 2004^31^

Developing amicable relations with PHC management is vital.^12^ In addition to PHC management, the community should be considered as stakeholders in the implementation of early communication intervention services. The role of grandmothers in the rural community should be valued as they act as indigenous gatekeepers of knowledge to younger generations regarding the development, care giving and well-being of women and children.^27^ Involving community stakeholders serves as an inclusive approach to planning and implementation and may increase interest, ownership and support for the early communication intervention programme. The researchers recommend that a rollout of functions should commence with primary prevention, which implies promotion and training activities (Figure 2). Against the background of poverty in infants’ care-giving environments, promotional activities for optimal early communication development may include the following: to raise parental awareness of the importance and their role in early communication and emergent literacy development for school readiness, the importance of first-language development in infants and young children; to promote safety to prevent injuries; to raise public awareness of available early communication intervention services; to advocate for enriched preschool education; and to facilitate the implementation of a language- and literacy-based preschool curriculum in nursery schools.^21^

#### Subtheme 6: The referral system in primary healthcare

The existing referral system for health-related cases in Ditsobotla subdistrict was not effective and involved a lengthy procedure of three operational organisational networks, since speech-language therapy and audiology teams at the secondary- and tertiary levels were located at facilities outside the subdistrict. The PHC personnel were uncertain when and to whom they should refer mothers with concerns regarding their children's hearing or communication development. Instead of referring directly to a speech-language therapist or audiologist, referrals were made through the doctor, who then had to refer to speech-language therapy or audiology services. It is within the extensive interdepartmental organisational network that a local referral system has to be adapted for improved service delivery.

Strategies suggested by participants for changing the referral system to improve service delivery included the following:

- Establish an effective interorganisational network to refer patients for early communication intervention services to tertiary-level services, such as diagnostic hearing testing.
- Collaborate with the PHC personnel to address the factors that are negatively influencing the referral system, such as transport problems and time delays.
- Develop context-specific referral systems, as unique needs and resources may be identified in a district.
- Provide in-service training when a referral framework is implemented in the subdistrict, so that PHC personnel can use the referral system successfully.
- Plan and manage early communication-intervention referral systems *within* the subdistrict.
- Establish effective communication between collaborators.
- Develop a comprehensive referral letter so that back-referral information can be recorded and sent to the referring healthcare professional.

Therefore, by providing feedback to the referring party, it helps to establish a relationship of trust and effective collaboration between the professionals and also helps to monitor patients on return visits to the clinician.

## Ethical considerations

The research study was approved by the Research Ethics and Proposal Committee of the Faculty of Humanities, University of Pretoria (Reference number: 21060038). Permission to conduct the research was granted by the North West Province Department of Health. All participants gave voluntary informed consent to participate in the study. Information obtained from the participants was treated confidentially and reported anonymously. All efforts were made to conduct the research according to the ethical principles of no harm or embarrassment to participants, veracity, non-discrimination and sensitivity to cultural- and language differences between participants and the researcher who collected the data.

## Trustworthiness

The researcher aimed to improve the trustworthiness of the study by improving the dependability, that is to say, the extent to which the results are reproducible. The researcher used data triangulation as well as detailed descriptions in order to get reproducible results.

### Validity

Strategies that were used to increase the validity of the instruments are as follows:

- Face validity was ensured as the format of the instruments corresponded with the aims of the data collection.
- Since the researcher interviewed the participants the content validity was improved as observations and conceptualisations were made during the interviews. The views of the PHC programme managers regarding the implementation of an early identification programme for infants at risk for developmental delays or disorders were explored in the semi-structured interviews.
- The instruments were developed in accordance with the study aims and relevant research findings in the field and were designed to be adaptable across cultural diversities, using constructs that remain the same across cultures, hence achieving construct validity.

### Reliability

With triangulation the reliability of the instruments is increased, as the data is collected by means of different instruments and results can therefore be compared. Furthermore, two separate groups of participants were used to investigate the identification methods and referral systems, whilst the researcher's own field notes also supported the data obtained in the interviews. The aim of the pilot testing of the instruments was to determine the reliability of the instruments. Each pilot testing made significant contributions, assisting the researcher in enhancing the reliability of the instruments. A second interpreter analysed the results obtained from the interviews with the participants in Group 1 and Group 2, which improved the reliability of interpretations.

## Discussion

Despite limited awareness of the benefits of early communication intervention, the lack of identification methods and ineffective referral systems in this impoverished rural subdistrict, PHC personnel (i.e. nurses in training, qualified nurses and sisters working in the PHC context) and PHC programme managers were interested in early communication intervention and had a positive attitude toward improving services.

### Primary healthcare personnel as valued partners

The PHC personnel at the different facilities can be instrumental in the early identification of infants and young children at risk of developmental delay or disorders. The level of entry for all patients into the health system is at the PHC facilities.^12^ PHC personnel address health problems directly or make referrals to a doctor or a specialised health professional. As the PHC personnel act as gatekeepers of the health care system,^28^ they need effective support in order to identify and refer infants and young children with risks as early as possible.

Despite their heavy workload, the PHC personnel and the PHC programme managers in Ditsobotla subdistrict responded positively to the concept of early communication intervention and wished to be part of the team. They were also agreeable regarding the implementation of different early communication intervention functions in increments at the facilities. They wanted to be consulted when selecting the functions to be implemented at each facility, as they are aware of the needs and the capacity of the human- and physical resources at the different facilities.

### Training programme for primary healthcare personnel

Based on results disclosing the *information needs* of the participants, components of such a training programme should include: Strategies to build trust, partnerships and effectively collaborate with other professionals; information on typical communication and emergent literacy development in infants and children; information sharing with parents; effective use of referral systems and identification methods; and the role of adults and environmental enrichment in early child development.

The results on self-identified training needs of the participants provided very clear suggestions to speech-language therapists and audiologists. Since the healthcare personnel in this rural area were unaware of early communication intervention and what it entails, community speech-language therapists and audiologists have an ideal opportunity to develop and present in-service training programmes to all PHC personnel and programme managers.

### Collaborative team approach

The results with regard to teamwork indicated that the participants wanted teamwork through dynamic collaboration and that they wanted to be consulted. Apart from the participants’ willingness to be involved in developing effective early communication intervention in the subdistrict, programme managers and trained volunteers are uniquely positioned to play effective roles. Doctors were identified as crucial role players in the early identification and referral of infants at risk^6^ and therefore need to be part of the early intervention team. The subdistrict may be characterised by poverty, but proved to be rich in human resources.

Much literature exists about building effective teams in early intervention. According to Rossetti,^19^ a transdisciplinary early communication intervention team approach is the preferred option. Different team members are dependent upon one another and share the knowledge, roles and responsibilities of negotiated functions. Shared information and roles between a speech-language therapist, an audiologist, PHC personnel and other health workers, if available, could therefore simplify the process of establishing a transdisciplinary early intervention team, as all have similar objectives to achieve. Teams may start small, as speech-language therapists and audiologists could be an effective transdisciplinary team of two professionals.^4^ Assessments and diagnosis may be discipline-specific professional roles, but screening, intervention and parent guidance may be shared roles.^4^

### Incremental implementation of early communication intervention functions

Van der Linde et al.^5^ already found that each of the PHC facilities in Ditsobotla subdistrict provided an opportunity and location to implement some of the early communication intervention functions as a means to reach infants at risk in a rural community. According to van der Linde,^6^ the assets and needs analysis indicated that all 20 PHC facilities in the subdistrict had permanent building structures, electricity and water supply and three clinics had been rebuilt or renovated recently. The implications were that early communication intervention services should be brought closer to the different communities, but that the full extent of preventative services and training, early identification, assessment and intervention of young children, parent training and diagnostic procedures could not be implemented at every PHC facility in the subdistrict at the time of data collection. Only three PHC facilities in Ditsobotla subdistrict had the capacity to support the full implementation of all early communication intervention functions.

Consistent with the incremental implementation process, the provision of early communication intervention functions at the different facilities will vary according to capacity and needs. The speech-language therapist and audiologist, in collaboration with the PHC personnel, clinic managers and PHC programme managers, should determine which functions should be implemented at the various facilities. The decisions of implementation should therefore be negotiated by an early communication intervention task team. If the speech-language therapist or audiologist cannot visit a specific clinic regularly, or when certain early communication intervention functions cannot be implemented at the facility due to lack of capacity, a referral has to be made to a nearby PHC facility. This process is known as ‘intradepartmental referral’.

The participants indicated that continuous monitoring of the implementation process is important. A needs assessment and environmental analysis should be conducted at regular intervals at each facility as the needs and capacity can change over time as the PHC facility develops. The needs assessment would also provide the clinician with insight into recurrent needs, the improvement of service delivery and the demand for services.

**FIGURE 3:**
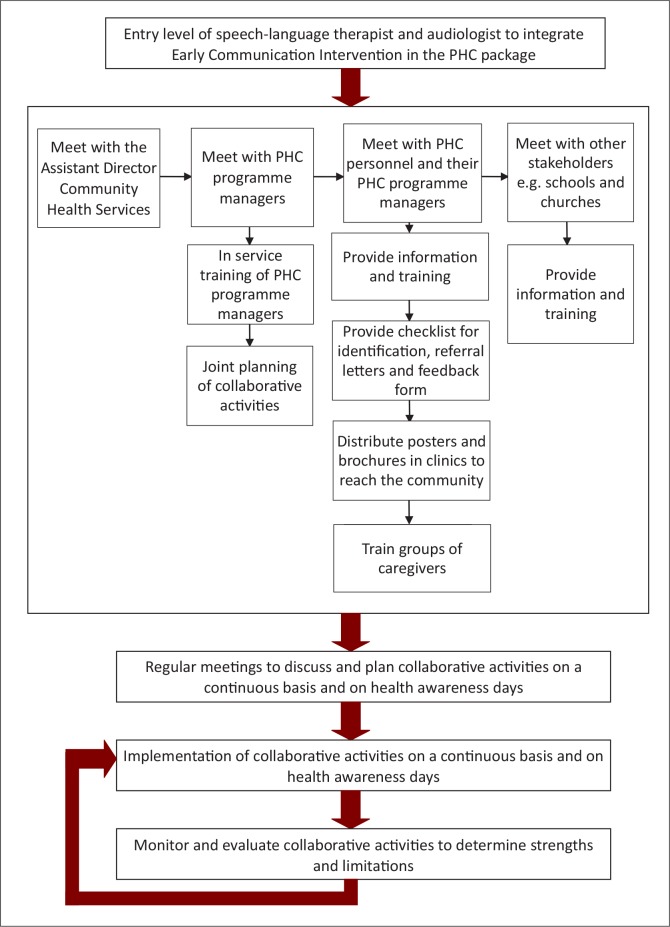
The process of integrating early communication intervention services into the Primary Healthcare Package.

An incremental rollout of the implementation of collaborative early communication intervention activities may involve the following:

- During the planning phase of collaborative activities the facilities need to be evaluated to determine whether there is capacity for successful implementation of the activities. Diverse activities may therefore be implemented at different facilities, according to capacity and needs.
- The incremental rollout of early communication intervention functions should not be considered as the only option, as once-off collaborative activities can be implemented at facilities with a limited capacity. The necessary arrangements should be made, such as allocation of space and training of volunteers to help with the implementation of the collaborative activities.
- Certain PHC programmes, such as Maternal, Child and Women's Health, should be utilised comprehensively, as many early communication intervention functions can be integrated in collaboration with these programmes.
- The participants indicated that early communication intervention should be introduced on four operational levels as indicated in Figure 3.

In summary, the four-level organisational framework (Figure 3) to implement early communication intervention services in increments at PHC facilities in the subdistrict was developed by the participatory process of the research study.

## Conclusion

Establishing sustainable early communication intervention in rural PHC communities is a challenge, as limitations influence the implementation of services in these contexts. The need for comprehensive early communication intervention in rural communities undoubtedly outweighs the limitations that may be experienced by clinicians. Implementation of early communication intervention in increments is a dynamic process that allows the clinician to monitor, add and adapt the functions implemented at the facilities, according to the needs of the specific community living around a PHC clinic or hospital. Early communication intervention functions should always be introduced by the collaboration with and training of partners in order to promote typical development, prevent disability and illness, screen and identify early, refer the family, conduct assessment, supply intervention, manage and evaluate programmes and continue with research. The results indicated that speech-language therapists, audiologists and PHC personnel may indeed *work together to address the problem* of early case finding and providing early communication intervention services to infants at risk. According to Olusanya et al.,^29^ reducing the burden of communication disorders should also be on the agenda of the many health priorities of the developing world.

A limitation of the research study is the exclusion of the general community in the subdistrict and therefore their needs, perceptions and suggestions regarding early communication intervention should be considered in future research. Collaborative partnerships with caregivers, professionals, PHC programme managers and volunteers, effective training programmes, extensive promotional activities, thoughtful application of methods and resources and an effective referral system are the building blocks required to reach the goal of accessible and affordable early communication intervention services in a community. Ultimately, an incremental implementation of early communication intervention may be integrated in the PHC package as part of a formalised programme. The impetus to expand early communication intervention services to rural communities lies in the evidence that ‘early intervention works’.^30^ This research should encourage clinicians to participate in this worthy endeavour to intervene as early as possible in the lives of all infants at risk.
